# The Molecular Mechanisms of Metformin’s Action on Blood Lipid Profile in Diabetic Patients

**DOI:** 10.3390/ijms27104635

**Published:** 2026-05-21

**Authors:** Agnieszka Dettlaff-Pokora, Julian Swierczynski

**Affiliations:** 1 Department of Biochemistry, Faculty of Medicine, Medical University of Gdansk, Debinki 1, 80-211 Gdansk, Poland; 2 Institute of Nursing and Medical Rescue, State University of Applied Sciences in Koszalin, 75-582 Koszalin, Poland

**Keywords:** metformin, serum cholesterol, serum triacylglycerol, AMPK, lipid metabolism

## Abstract

In this paper, we review the literature regarding metformin’s action on blood lipid concentrations in metformin-treated diabetic patients. Published data indicate that metformin reduces serum total cholesterol (T-C), LDL-cholesterol (LDL-C) and triacylglycerol (TAG) concentrations and raises serum HDL-cholesterol (HDL-C) concentrations in diabetic patients. The beneficial effect of metformin on serum lipid profiles in diabetic patients can result from (a) its action on AMP-activated protein kinase, which inhibits lipogenesis and cholesterol synthesis and stimulates fatty acid oxidation; (b) decreased plasma TAG concentrations, via promoting VLDL-TAG clearance by brown adipose tissue; (c) the inhibition of nuclear factor erythroid 2-related factor 2 (*Nrf2*) gene expression, affecting lipid profile in diabetic patients; (d) the inhibition of the expression of genes encoding proprotein convertase subtilisin/kexin 9 (PCSK9) and lipogenic enzymes; (e) the downregulation of carbohydrate-response element-binding protein (ChREBP), which affects liver TAG and cholesterol synthesis from acetate formed by gut microbiota; (f) the inhibition of angiopoietin-like 3 protein (ANGPTL3) gene expression, and consequent effects on plasma TAG concentrations; (g) the activation of AMPK, which inhibits LXRα activity; and (h) reverse cholesterol transport. In conclusion, one can assume that beyond its primary antihyperglycemic effect, metformin exerts pleiotropic effects that modulate lipid metabolism and blood lipid profile in T2D patients. These beneficial effects of metformin on blood lipid profile may play a role in the reduction in cardiovascular risk in diabetic patients.

## 1. Introduction

**Figure 1:**
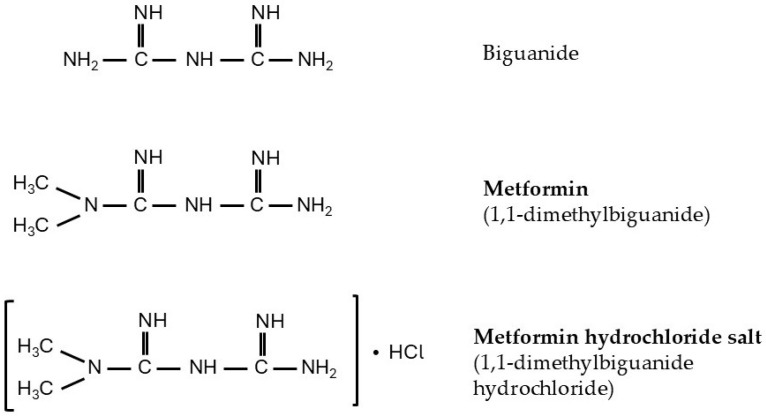
Chemical structures of biguanide, metformin (1,1 dimetylbiguanide) and metformin hydrochloride salt (1,1-dimethylbiguanide hydrochloride).

Oral metformin (1,1-dimethylbiguanide and its hydrochloride salt), well tolerated by the majority of patients, is a first-line hypoglycemic drug in the treatment of type 2 diabetes (T2D). It is also a relatively cheap and effective antihyperglycemic drug. The glycemic response exerted by metformin in patients is variable and depends on their glycemic and lipemic status. Some patients with T2D respond very well, whereas others respond considerably less or even show no benefit [1]. The chemical structure of biguanide, and its main derivatives 1,1-dimetylbiguanide (IUPAC name—N,N-dimethylimidodicarbonimidic diamide) and 1,1-dimethylbiguanide hydrochloride (IUPAC name—1-carbamimidamido-N,Ndimethylmethanimidamide hydrochloride), are presented in Figure 1.

Metformin is used daily by more than 200 million patients with T2D worldwide as monotherapy or in combination with other antidiabetic drugs [2,3]. The drug has only a slight effect on circulating glucose concentration in healthy subjects; thus, metformin is sometimes called an antihyperglycemic rather than a hypoglycemic agent [4]. The drug also has several other effects, including improved serum lipid profile, modest body weight loss and enhanced insulin sensitivity, all of which contribute, at least in part, to its antidiabetic action [5,6,7,8,9]. Metformin may also have other potential therapeutic properties beyond antidiabetic effects, including a reduction in cardiovascular disease and mortality [10] and anti-inflammatory and antioxidant properties [11,12]. Some authors, based on epidemiological analysis, basic science and clinical observations, have suggested the effectiveness of metformin in targeting some age-related human diseases [11,13]. It was also proposed that due to its anti-aging [11,13], anti-inflammatory [14], and antiviral [11,15] properties, metformin could have a protective and therapeutic effect against COVID-19 [15].

Additionally, it has been suggested that the use of metformin in T2D patients was associated with lower risk of incidence of some cancers and mortality [16,17,18]. Some authors proposed that the antitumor effect of metformin is associated with an increase in AMPK activity caused by the drug [19,20]. Recently published data suggest that prostate cancer patients with low expression of the NKX3.1 gene (an androgen-regulated gene encoding transcription factor and a negative regulator of epithelial growth in prostate tumors) [21] may also benefit from metformin treatment [22]. One can conclude that the primary clinical benefits of metformin result from its antihyperglycemic effects, which secondarily reduce the risk of other above-mentioned pathologies. However, it is very likely that some health benefits are not directly associated with the effects of metformin on blood glucose concentrations. In this review, we mostly focus on the effects of metformin on lipid metabolism and lipid blood profile. We especially concentrated on the molecular mechanisms underlying metformin’s lipid-lowering effects in T2D patients.

## 2. Metformin Absorption and Disposal

**Figure 2:**
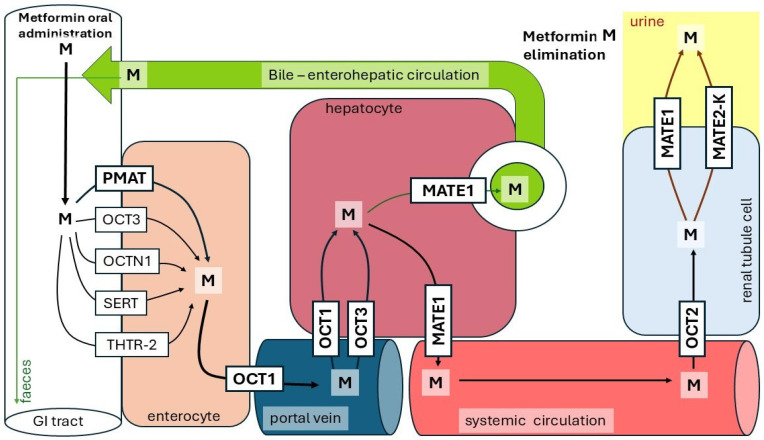
Metformin absorption and elimination. Key roles are played by the proximal small intestine (enterocyte), liver (hepatocyte) and kidney (renal tubule cell). Abbreviations: Metformin (M), Plasma membrane monoamine transporter (PMAT), Organic cation transporter 1 (OCT1), Organic cation transporter 2 (OCT2), Organic cation transporter 3 (OCT3), Carnitine/organic cation transporter (OCTN1), Multi-drug and toxin extrusion-1 transporter (MATE-1), Multi-drug and toxin extrusion2-K transporter (MATE2-K), Serotonin reuptake transporter (SERT), Human thiamine transporter (THTR-2).

Metformin is usually taken orally by diabetic patients as the hydrochloride salt (1,1-dimethylbiguanide hydrochloride, see Figure 1) in conventional immediate-release or modified-release tablet form. Diabetic patients usually receive 1 to 1.5 g of metformin per day, divided into two or three doses. The maximal approved daily dose of the drug is 2.5 g (or, more precisely, 35 mg per kg body weight) [23,24]. After oral intake of metformin at 0.5–1.5 g per day by healthy volunteers, absorption of the drug was 70–80%, and absolute oral bioavailability was estimated to be 33–55% [3]. In the human body, under physiological conditions, metformin exists mainly in an organic cation form. After being swallowed, metformin absorption takes place mainly in the proximal small intestine (mainly in the duodenum) [25,26,27]. The drug enters intestinal epithelial cells through the luminal side, mainly via the plasma membrane monoamine transporter (PMAT), a polyspecific organic cation transporter encoded by the solute carrier family 29 member 4 (SLC29A4) gene [28]. In the human intestine, metformin has an apparent Km of approximately 1.3 mM for PMAT [29]. Moreover, it has been found that the acidic environment in the intestinal lumen can promote PMAT-dependent metformin uptake [30]. Some other polyspecific organic cation transporters are also involved in intestinal metformin absorption. For instance, the organic cation transporter 3 (OCT3), present in the brush border membrane of enterocytes, plays an important role in metformin absorption [31]. Moreover, carnitine/organic cation transporter (OCTN1) encoded by SLC22A4 [32], serotonin reuptake transporter (SERT) encoded by SLC6A4 [33], and the human thiamine transporter (THTR-2) encoded by SLC19A3 [34] are also involved in intestinal metformin absorption. The results presented above indicate that various polyspecific organic cation transporters present on the intestinal brush border are involved in the transport of metformin from the intestinal lumen into enterocytes (Figure 2). Intracellular metformin next crosses the basolateral membrane of intestinal cells through organic cation transporter 1 (OCT1) encoded by the SLC22A1 [28], reaching portal circulation [35,36]. The metformin, via portal vein, is then delivered to the liver (Figure 2). Plasma obtained from the portal-vein blood of animals treated with the therapeutic dose of metformin contains 40–70 µM of the drug [37].

In the human liver, the main organ of metformin action, which mainly reduces glucose synthesis via inhibition of gluconeogenesis [38], and affects lipid metabolism, OCT1 and OCT3 play an important role in metformin transport into hepatocytes [39,40] (Figure 2). OCT1 is highly polymorphic in ethnically diverse populations and mediates differences in transport functions [41,42]. Thus, some authors proposed that OCT1 may influence the hepatic elimination and efficacy of metformin, which can be responsible for interindividual variations in the drug action [43,44]. However, the role of OCT1 in the pharmacogenetics of metformin response and intolerance still remains controversial. For instance, some authors indicated that they had found no significant effect of SLC22A1 polymorphism on metformin efficacy [45].

Experiments performed on primary rat hepatocytes indicate that the Km for metformin uptake is approx. 220 µM, which is significantly higher (3- to 5-fold higher) than the drug concentration in the portal vein [46]. It is widely accepted that intracellular metformin is not metabolized (or very slowly metabolized under physiological conditions—see below) in the liver, but it is excreted from hepatocyte by the multi-drug and toxin extrusion-1 (MATE-1) transporter present in the basolateral membrane (also called sinusoidal membrane), and in the canalicular membrane of hepatocytes, to the systemic circulation and the bile canaliculi, respectively [37] (Figure 2). In the systemic circulation, the plasma concentration of the drug is (10–40 µM) lower than in the portal vein [37,47]. Comparing the concentration of metformin in the portal vein (40–70 µM) and systemic circulation (10–40 µM) (Figure 2) and keeping in mind that metformin is not metabolized (or is very slowly metabolized under physiological conditions) in the liver, one can conclude that significant amounts of the drug are excreted to the bile and then to the alimentary tract. Finally, significant amounts of metformin used by diabetic patients can be detected in feces.

However, some data indicate that rat liver can metabolize metformin and that CYP2C11, CYP2D1 and CYP3A1/2 are involved in this process [48]. Moreover, it has been shown that inducers of the above-mentioned CYPs (for instance, dexamethasone, which is often used by patients to treat some pathologies), through induction of CYPs, significantly increase metformin metabolism [48]. Thus, the authors of this paper postulate that significant changes in metformin pharmacokinetics may occur in diseases associated with an increase or decrease in the activity of CYP2C11, CYP2D1 or CYP3A1/2 [48]. However, it cannot be guaranteed that the pharmacokinetics of metformin in the liver of a rat and the liver of a human are similar.

The main clearance pathway of metformin is renal elimination [25,49,50]. Thus, in addition to the intestine and liver, the kidney plays an important role in the pharmacokinetics of metformin. In general, metformin is absorbed from the systemic circulation into renal epithelial cells (renal tubule cells) and then excreted into urine. It seems that organic cation transporter 2 (OCT2), expressed in the basolateral membrane of human renal tubule cells, plays a key role in entry of metformin into the tubule cells, whereas the multi-drug and toxin extrusion-1 (MATE-1) and the multi-drug and toxin extrusion2-K transporter (MATE2-K), present in the brush-border membrane, mediate the secretion of the drug into lumen of proximal tubules (simply into urine) [51,52,53,54,55] (Figure 2). MATE2-K exhibits a kidney-specific expression, whereas human MATE-1 is highly expressed in many organs, including kidney, liver, skeletal muscle, adrenal gland and other organs [56]. In humans, OCT2 (SLC22A2), together with MATE-1 (SLC47A1) and MATE2-K (SLC47A2), play a key role in the metformin renal elimination. After intravenous administration of metformin at doses of 0.25–1.0 g to healthy volunteers, 80–100% of the dose was excreted in urine via renal tubular secretion [3].

OCT3 is present in cells of several human and animal organs, including white adipose tissue, skeletal muscle, lung, prostate, and salivary glands [57,58,59]. This suggests that OCT3 may play an important role in metformin tissue distribution.

## 3. Metformin as an Antihyperglycemic Drug

In the last few years, metformin has been the most-prescribed anti-diabetic drug used worldwide for the treatment of T2D [60]. The antidiabetic effect of metformin is a reduction in liver glucose output, mainly due to inhibition of the rate of gluconeogenesis [61,62,63,64,65]. However, the molecular mechanism of metformin’s action on gluconeogenesis has not yet been fully established, and the possible mechanisms of the drug’s action in this process are still debated. Several mechanisms have been proposed to explain the molecular mechanism of metformin’s action on liver glucose production [24,66,67,68,69,70,71,72]. Since the glucoregulatory mechanism of metformin’s action in T2D patients has been extensively reviewed in the last few years [11,24,69,71,72,73], in this review, we focus on the effects of metformin on lipid metabolism and lipid blood profile. The effect of metformin on glucose metabolism in T2D patients is only briefly presented below.

One of the most extensively studied molecular mechanisms of metformin action is associated with the inhibition of mitochondrial respiratory chain complex I in the liver [24]. In general, metformin action on mitochondrial respiratory chain complex I leads to increases in (a) the redox state (increase NADH:NAD^+^ ratio by reducing NADH oxidation) and (b) intracellular AMP and ADP levels (precisely, AMP:ATP and ADP:ATP ratios) [24]. Both elevated NADH:NAD^+^ ratio and the changes in the AMP:ATP and ADP:ATP ratios lead to inhibition of lactate and glycerol conversion into glucose, resulting in a decrease in serum glucose concentration [72]. Increases in the AMP:ATP and ADP:ATP ratios lead to activation of AMPK, which in turn catalyzes phosphorylation of several target proteins controlling many anabolic and catabolic processes, including gluconeogenesis [74,75,76,77]. Therefore, it has been proposed that metformin can inhibit glucose production in gluconeogenesis via activation of AMPK [24]. However, metformin can inhibit gluconeogenesis in the mouse liver independently of the liver kinase B1 (LKB1)-AMPK pathway through a decrease in liver energy state [67]. Moreover, metformin, via increases in the intracellular AMP levels, inhibits adenylate cyclase, reducing the effect of glucagon on cAMP concentration and protein kinase A (PKA) activity, and consequently leading to inhibition of gluconeogenesis [78]. Recent studies indicate that metformin activates AMPK via the lysosomal pathway [70,79,80,81]. A key role in this process is played by presenilin enhancer 2 (PEN2), which binds metformin and forms a complex with ATP6AP1, a subunit of the v-ATPase present in the lysosomal membrane [70,80,81]. All of these events lead to the suppression of v-ATP-ase and activation of AMPK, without effects on the AMP:ATP and ADP:ATP ratios [70,80,81]. Moreover, metformin can directly inhibit mitochondrial glycerol 3-phosphate dehydrogenase, leading to (a) increased cytosolic NADH:NAD^+^ ratio and consequently to inhibition of gluconeogenesis from lactate and glycerol; and (b) inhibition of the activity of the glycerol 3-phosphate shuttle, which is responsible for hydrogen transfer from cytosolic NADH into mitochondria [72]. Metformin also increases redox state via an increase in the reduced:oxidized glutathione (GSH:GSSG) ratio, leading to suppression of genes encoding enzymes involved in gluconeogenesis [72]. Moreover, metformin is able to inhibit complex IV of the mitochondrial respiratory chain and consequently to prompt an increase in the mitochondrial and cytosolic NADH:NAD^+^ ratio [72].

In addition to the liver, the skeletal muscle and intestine play some role in the lowering of circulating glucose concentration by metformin [24]. After metformin administration, an increase in peripheral glucose disposal was observed. This was mainly due to an increase in non-oxidative glucose disposal into skeletal muscle [62]. Furthermore, in skeletal muscle, metformin increases insulin-stimulated glucose uptake, which consequently results in a decrease in blood glucose concentration [24]. Possibly, this effect is secondary to improved glycemic control and a reduction in glucose toxicity [82]. In the intestine, metformin exerts alterations in (a) microbiome composition, which is associated with improved intestinal barrier integrity, increased production of short chain fatty acid, and regulation of bile acid metabolism; (b) intestinal glucose uptake; (c) secretion of some incretin hormones (for instance, GLP-1-glucagon-like peptide) and GDF15 (growth differentiation factor 15), as well as delayed gastric emptying and altered enterocyte glucose metabolism [24,72,83,84]. Metformin treatment increases the levels of GDF15 mRNA, not only in the intestine, but also in the liver, skeletal muscle and kidney of mice fed a high-fat diet [9,85,86]. The release of GDF15 from the intestine after metformin treatment was also found in humans [87]. Moreover, it has been shown that increased GDF15 blood concentrations have been associated with body-mass loss and reduced appetite, both in humans and mice [88]. However, other authors did not confirm that GDF 15 is necessary for the body-mass lowering effect of the drug [85].

Very recently, it has been shown that mice lacking Ras—related protein 1 (which plays a role in regulation of many cellular processes [89], including energy balance and glucose homeostasis [90] in forebrain neurons, did not respond to low doses of metformin, even though they still responded to the other antidiabetic drugs, including insulin, GLP-1 agonists, and SGLT2 inhibitors [91]. Moreover, it has been shown that (a) injection of metformin directly into the mouse brain at very low doses decreased blood glucose concentrations, and (b) in mice brain slices, metformin causes depolarization of SF1 neurons, although only when Rap 1 is present [91]. These results suggest that low-dose metformin requires brain Rap1 for its antidiabetic action [91].

The results discussed above indicate that the antihyperglycemic effects of metformin are due to its pleiotropic action, not only in the liver, but also in skeletal muscle, intestine (and its microbiota) and brain.

## 4. Metformin as a Blood Lipid-Lowering Agent in Patients with T2D

T2D patients usually have (a) an elevated blood concentration of TAG, (b) a reduced blood concentration of HDL-C, and (c) a high small-dense LDL-particle concentration, a lipid profile known as diabetic dyslipidemia [92,93]. It is generally believed that this lipid pattern is particularly atherogenic. Many experts consider T2D to be a fat disease, whereas type 1 diabetes is considered to be a sugar disease [93]. Dyslipidemia is an important risk factor for the development of macrovascular and microvascular diseases in T2D patients [94,95,96]. Clinical observation indicates that metformin alone significantly reduced plasma LDL-C concentrations after treatment of T2D patients [97,98,99]. Moreover, it has also been shown that patients who were supplied with metformin had significant decreases, not only in plasma T-C and LDL-C, but also in TAG concentrations [4,100,101,102]. A meta-analysis published more than 20 years ago suggested that metformin reduces blood T-C and LDL-C concentrations in patients with T2D, but the reductions are relatively small [103]. In contrast, more recently published data indicate that metformin significantly reduces serum LDL-C and TAG concentrations and raises serum HDL-C concentrations in patients with T2D [104,105,106]. Moreover, a relatively low dose of metformin causes a decrease in plasma non-HDL-C lipids in patients with prediabetes [107]. A meta-analysis has shown that metformin decreases the concentrations of T-C, LDL-C and TAG in the blood of non-diabetic patients [108]. Another meta-analysis showed that metformin treatment of subjects at risk for diabetes also improves lipid profile [105]. Moreover, it has been shown that atorvastatin is more effective in reducing the atherogenic lipid parameter in metformin responders, compared to nonresponders, in newly diagnosed patients with T2D [109]. Randomized controlled trials showed the beneficial effects of metformin + DPP-4 inhibitor and metformin + GLP agonist on T-C, LDL-C, and HDL-C [110]. Moreover, metformin monotherapy (a) reduces the risk of macrovascular disease in patients with T2D [111] and (b) was associated with a lower risk of cardiovascular-related morbidity and mortality [112]. The data presented above indicate that metformin monotherapy and metformin combined with statins, DPP-4 or GLP agonist decrease plasma T-C, LDL-C and TAG concentrations. Some studies indicated an increase in HDL-C concentrations in T2D patients treated with metformin. These beneficial effects of metformin on lipid profile are associated with a lower risk of cardiovascular diseases. However, inconsistent data regarding the effect of metformin on lipid profiles in T2D patients has also been reported. For instance, Buse et al. [113], based on the data published from 1966 to 2002, concluded that metformin reduced T-C, but the drug’s effect on LDL-C, TAG and HDL-C in T2D patients was inconclusive. No effect of metformin on T-C, non-HDL-C and HDL-C in the plasma of T2D patients was reported [114]. The same group showed that metformin treatment was associated with a decrease in plasma VLDL-triacylglycerol concentration [114]. In contrast, Gormsen et al. [115] reported that metformin treatment had no effect on plasma VLDL-triacylglycerol concentration in T2D patients. Another study showed no effect of metformin on lipid profile in T2D patients [116]. A meta-analysis of randomized controlled trials performed on data published from 2010 to 2020 showed that T-C, LDL-C, and TAG are decreased in patients with T2D who are treated with metformin [102]. The differing results presented above might be due to several factors, including metformin dose, duration of treatment and diet consumed by patients. In general, the data regarding the effect of metformin on plasma lipid profile is inconsistent; in most studies, there were at least moderate improvements in plasma TAG, T-C, LDL-C, non-HDL-C, and HDL-C concentrations.

Besides lowering plasma lipid concentrations, metformin reduces body weight, which is associated with the redistribution of fat from visceral to subcutaneous depots, and prevents vascular complications [4]. Moreover, in patients with non-alcoholic fatty liver disease (NAFLD) and non-alcoholic steatohepatitis (NASH), metformin improved liver function [117,118,119]. It has also been shown that metformin can be useful for improving dyslipidemia in non-diabetic patients. Moreover, it has been shown that an elevated concentration of LDL-C, induced by antipsychotic drugs used by patients with schizophrenia (having physiological blood glucose concentration), was reduced by metformin [120,121,122]. These data suggest that the effect of metformin on dyslipidemia in diabetic patients could also be independent, at least in part, of the improvement in T2D. The beneficial effect of metformin on blood lipid profile in diabetic patients was confirmed in experimental animal models [123,124,125].

The data presented above clearly indicate that metformin improves dyslipidemia (a) in non-diabetic patients, (b) in subjects at risk for diabetes, (c) in patients with T2D, and (d) in some pathologies not directly associated with diabetes (non-diabetic patients). Furthermore, it has been reported that in T2D patients, metformin enhances the lipid-lowering efficacy of some statins [109]. Therefore, metformin not only controls hyperglycemia, but also dyslipidemia, reducing cardiovascular risk in patients with T2D. It seems that the beneficial effects of metformin on lipid metabolism and blood lipid profile are important in T2D patients; however, this is often overlooked by medical clinicians. Despite its potential in lowering blood lipid concentrations, the molecular mechanism of metformin’s action on lipid metabolism and consequently on blood lipid concentrations is still not clear. The potential mechanisms of metformin action on lipid metabolism and blood lipid profile, especially in T2D patients, are presented below.

### 4.1. Potential Role of AMP-Activated Protein Kinase Activation by Metformin in the Regulation of Lipid Metabolism

More than 20 years ago, it was found that metformin activates liver AMP-activated protein kinase (AMPK) [126]. AMPK is heterotrimeric protein consisting of (a) catalytic α subunit, which has two isoforms, α1 and α2; (b) subunit β (isoforms: β1 and β2), which act as a scaffold binding to other subunits of AMPK; and (c) subunit γ (isoforms: γ1, γ2 and γ3) binding of AMP and/or ADP to specific domains [76,77,78]. In the human liver, the predominant AMPK complex consists of α1, β2 and γ1 subunits (AMPKα1,β2γ1). AMPK is a conserved serine/threonine protein kinase [76,78,127], protecting cellular function under energy restricted conditions [76,77,127], including regulation of carbohydrate and lipid metabolism [66,76]. Activation of AMPK activity involves (a) allosteric activation by AMP (and also by ADP) and (b) phosphorylation of α subunit on threonine 172 (Thr 172) [77,128]. The phosphorylation of α subunit on Thr 172 is catalyzed by serine/threonine kinase 11 (STK 11), also known as Liver kinase B1 (LKB 1), in response to energy deficits in the cells, and by Ca^2+^/calmodulin dependent protein kinase 2 (CaMKK2) in response to increased intracellular Ca^2+^ concentrations [77,128]. AMP is binding to γ subunits on a specific site of the AMPK complex, which causes conformational changes that allosterically activate the enzyme and inhibit the dephosphorylation of Thr 172 of the catalytic α subunit [72,114]. Phosphorylated enzyme is allosterically stimulated by AMP or ADP [77,128]. Therefore, it is generally believed that AMPK is activated by an increase in the intracellular AMP:ATP and ADP:ATP ratios resulting from an imbalance between ATP synthesis and utilization by several processes, including carbohydrate and lipid metabolism [77,128]. It is generally accepted that metformin does not directly activate AMPK and/or LKB1 [68,77,128]. AMPK activation by metformin is secondary to the drug’s effect on mitochondria, especially with respect to the inhibition of mitochondrial respiratory chain complex I [68,72,77,126,128], which leads to increased AMP:ATP and ADP:ATP ratios [77,128].

Activated AMPK catalyzes phosphorylation of its downstream substrates, to reduce ATP-consuming anabolic processes, including fatty acid and cholesterol synthesis, whereas it stimulates ATP-producing catabolic processes such as fatty acid oxidation [77,126,128]. Active AMPK phosphorylates (a) acetyl-CoA carboxylase isoforms 1 and 2 (ACC1 and ACC2, respectively) and (b) 3-hydroxy-3-methylglutaryl-CoA reductase (HMG-CoA reductase; also known as β-hydroxy-β-methylglutaryl-CoA reductase) [129]. ACC1 (in active-dephosphorylated form) produces malonyl-CoA (according to the reaction acetyl-CoA + HCO_3_^−^ + ATP → malonyl-CoA + ADP + Pi), and plays a key role in *de novo* fatty acid synthesis, and the biosynthesis of substrates for TAG and phospholipids, mainly in the liver and adipose tissue. Malonyl-CoA produced by skeletal muscle ACC2 is an allosteric inhibitor of carnitine palmitoyl-CoA transferase 1 (CPT1), which catalyzes conversion of palmitoyl-CoA (or other long chain acyl-CoA) to palmitoylcarnitine (or corresponding long-chain acyl-carnitine) according to the reaction acyl CoA + carnitine + ATP → acyl-carnitine + HS-CoA + AMP + PPi). This enzyme plays a key role in acyl-CoA transport into mitochondria, and consequently in fatty acid oxidation, mainly in heart and skeletal muscles, the kidney and the liver. Metformin through stimulation of AMPK, which in turn phosphorylates and consequently inhibits both ACC1 and ACC2 [126,128,130], reduces malonyl-CoA synthesis.

**Figure 3:**
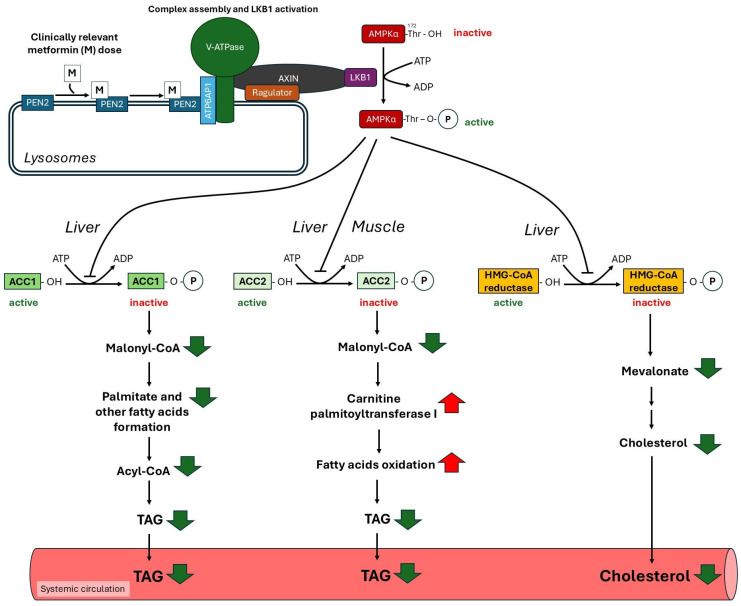
Potential role of AMPK-activated protein kinase (AMPK) activation by metformin in decreased blood cholesterol and triacylglycerol (TAG) concentrations. AMPK, activated by metformin, inactivates via phosphorylation of acetyl-CoA carboxylase 1 and 2 (ACC1 and ACC2) and 3-hydroxy-3-methylglutaryl-CoA reductase (HMG-CoA reductase) in liver and muscle, which finally leads to decreases in blood TAG and cholesterol concentrations.

Reduced concentration of intracellular malonyl-CoA in liver and adipose tissue results in inhibition of fatty acid synthesis [126,128], whereas reduced concentration of malonyl-CoA (allosteric inhibitor of CPT1) in heart and skeletal muscle due to inhibition of ACC2 leads to increases in fatty acid oxidation and ATP production [126,128] (Figure 3). Moreover, activation of AMPK by metformin also inhibits expression of the gene encoding liver sterol regulatory element-binding protein 1c (SREBP-1c}, which plays a key role in the regulation of lipogenic enzymes gene expression, including fatty acid synthase (FASN) [112,118,119,120,126,131,132,133]. Via downregulation of lipogenic enzymes, including ACC, metformin may inhibit liver lipid biosynthesis and consequently can decrease circulating lipid concentrations. Considering that AMPK inhibits expression of the gene encoding FASN and ACC via direct phosphorylation of carbohydrate response element binding protein (ChREBP) [134], one can conclude that metformin in this way may also suppress lipogenesis. In addition, metformin, via activation of AMPK, inhibits gene expression encoding stearoyl-CoA desaturase 1 (SCD1), a rate-limiting enzyme responsible for biosynthesis of monounsaturated fatty acids [135,136].

HMG-CoA reductase, which catalyzes (in active—dephosphorylated form) conversion of HMG-CoA to mevalonate (according to the reaction HMG-CoA + 2NADPH + 2H^+^ → mevalonate + 2 NADP^+^ + HS-CoA) is the rate-limiting enzyme in cholesterol biosynthesis [124]. Similar to ACC, phosphorylation of HMG-CoA reductase by AMPK leads to inhibition of the enzyme activity, and consequently, to reduction of cholesterol biosynthesis [137] (Figure 3).

The information presented so far indicates that metformin, via activation of AMPK, may significantly (a) inhibit liver lipogenesis and cholesterol synthesis, and (b) increase fatty acid oxidation in the liver, skeletal muscle and heart. These effects are likely to contribute to metformin’s ability to reduce the blood triacylglycerol and cholesterol concentrations in T2D patients treated with the drug (Figure 3).

The view that activation of AMPK by an increase in intracellular AMP:ATP and ADP:ATP ratios results from inhibition of complex I of the mitochondrial respiratory chain is widely accepted [28,125]. However, inhibition of respiratory chain complex I requires relatively high (2–5 mM), suprapharmacological, metformin concentrations [138]. Such concentrations cannot be achieved during daily treatment of T2D patients with the standard dose of metformin [75]. Therefore, the standard dose of the drug taken by T2D patients may not be sufficient to increase the AMP:ATP and ADP:ATP ratios, and consequently to activate AMPK [139,140]. Zhang et al. found that a low dose of metformin (that is, a clinically relevant dose) inhibits the lysosomal pump v-ATP-ase which plays a key role in AMPK activation after glucose starvation [141]. It has also been shown that metformin can promote the translocation of AXIN/LKB1 onto the surface of lysosome to form v-ATP-ase-Ragulator-AXIN/LKB1-AMPK complex, leading to AMPK activation [79,142,143]. More recently, it has been reported that a clinically relevant dose of metformin activates the lysosomal AMPK via PEN2 (presenilin enhancer 2) [70]. PEN2 is a component of complex γ-secretase, an enzyme cleaving various type I transmembrane proteins [144,145]. PEN2 and anterior pharynx defective 1 (APH-1) interact directly with each other and with presenilin. These interactions are an important step in γ-secretase complex assembly [146]. Ma et al. found that lysosomal PEN2 binds metformin and that the complex of metformin–PEN2 is recruited to ATP6AP1 (ATPase H+ Transporting Accessory Protein 1) of the v-ATP-ase complex, which next activates lysosomal AMPK, without increasing AMP:ATP and ADP:ATP ratios [70]. Based on the above presented data, Ma et al. [70] proposed that metformin, at a clinically relevant dose, via binding to PEN2, promotes the formation of the metformin-PEN2-ATP6AP1-v-ATPase-Ragulator-AXIN-LKB1 complex, which independently of AMP:ATP and ADP:ATP ratios, stimulates AMPK. Thus, one can conclude that activation of lysosomal AMPK via binding of metformin to PEN2 plays a crucial role in the regulation of lipogenesis, cholesterol synthesis and fatty acid oxidation in T2D patients (Figure 3). However, it is not excluded that at suprapharmacological concentrations of metformin, increases in AMP:ATP and ADP:ATP ratios (due to inhibition of mitochondrial function) also take place.

It seems that lysosomal AMPK activation by metformin through binding of the drug to PEN2 also plays an important role in lipid metabolism via an increase in glucagon-like peptide (GLP-1) secretion in the intestine [72], which downregulates SREBP-1c in rat liver and reduces hepatic triacylglycerol deposition [147]. Furthermore, it also has been reported that GLP-1 analogs (for instance Lira) may also improve lipid metabolism via (a) downregulation of gene expression encoding FASN and ACC1, and (b) by increasing fatty acid oxidation in the mouse liver [147].

Several studies indicate that GLP-1 is also closely associated with cholesterol metabolism (for details see Ref. [147]. For instance, it has been shown that GLP-1 significantly increases ATP-binding cassette A1 (ABCA1) and ATP-binding cassette G1 (ABCG1) levels, which are associated with decreases in the intracellular level of cholesterol in macrophages [147]. In this manner, GLP-1 inhibits the transformation of macrophages into foam cells and prevents the development of atherosclerosis [147]. Exendin-4, another analog of GLP-1, reduces the level of liver sterol regulatory element binding protein 2 (SREBP2), which leads to inhibition of cholesterol biosynthesis and serum cholesterol concentration in mice [148]. A clinical study demonstrated that GLP-1 and glucose-dependent insulinotropic peptide (GIP) dual receptor agonist significantly reduced triacylglycerol and total cholesterol concentration in human blood [149]. GLP1/GIP dual receptor agonist also significantly reduces total cholesterol, LDL-C and triacylglycerol concentrations, whereas it increases HDL-C concentration [150].

The information presented so far suggests that a clinically relevant dose of metformin used to treat T2D patients, possibly via lysosomal AMPK activation, can exert a beneficial effect on blood lipid profile (Figure 3), and can prevent the development of atherosclerosis.

The data presented above completely match the suggestion that metformin exerts a direct antiatherogenic action. This antiatherogenic action of metformin may be independent of its effect on blood glucose concentration [151]. Thus, stimulation of lysosomal AMPK by metformin may be an important action against atherosclerosis, given its role in lipid metabolism.

### 4.2. Metformin Decreases Plasma Triacylglycerol Concentration via Promoting VLDL-Triacylglycerol Clearance by Brown Adipose Tissue (BAT)

The molecular mechanism underlying the effect of metformin on lipid metabolism was studied by using transgenic mice (APOE3-LeidenCETP)—an experimental model of human-like lipoprotein metabolism [152]. Geerling and coworkers [68], using this experimental model, have shown that metformin lowered plasma T-C and TAG concentrations. This effect was mainly caused by decreased plasma VLDL-TAG concentrations [68]. Surprisingly, in this experimental model, metformin did not affect liver VLDL-TAG production, VLDL and liver lipid composition, whereas significantly increased VLDL-TAG clearance by brown adipose tissue (BAT) was found [68]. BAT mass and lipid droplet content in this tissue were significantly decreased in metformin-treated transgenic mice [68]. Moreover, AMPK and hormone-sensitive lipase (HSL) activities, as well as mitochondrial content, were also significantly increased in BAT of transgenic mice treated with metformin [68]. Additionally, it has been found that therapeutic concentrations of metformin increased AMPK and HSL activities as well as lipolysis in T37i differentiated brown adipocytes [68]. Based on these data, the authors proposed that metformin stimulates fatty acid oxidation in BAT as a result of increased lipolysis (due to an increase in HSL) and mitochondrial content [68]. The information presented so far suggests that metformin could exert a beneficial effect on circulating lipids via lowering plasma TAG concentrations. In this process, crucial roles are played by (a) a selective BAT-mediated increase in VLDL-TAG uptake, (b) an increase in BAT lipolysis and (c) an increase in the oxidation of fatty acids.

Several studies suggest that functionally active BAT is also present in the human body and the amount of this tissue is inversely correlated with body mass index [153,154,155,156,157]. Moreover, it has been reported that human BAT displayed fatty acid uptake upon exposure to cold [155,158]. Thus, it is tempting to speculate that the beneficial effect of metformin on lipid profile in T2D patients treated with the drug is also associated, at least in part, with the promotion of VLDL clearance by BAT.

### 4.3. Metformin, via an Increase in the Nuclear Factor Erythroid 2-Related Factor 2 (Nrf2) Expression, Could Exert a Beneficial Effect on Lipid Profile in Diabetic Patients

**Figure 4:**
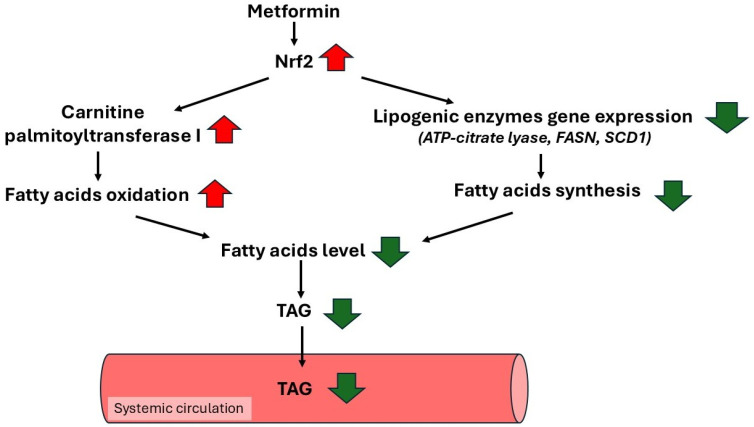
Metformin increases the nuclear factor erythroid 2-related factor 2 (Nrf2) transcription. That leads to limitation of fatty acid synthesis (due to inhibition of lipogenic enzymes’ gene expression), and an increase in fatty acid oxidation (due to activation of carnitine palmitoyltransferase I gene expression). Ultimately, less TAG is formed.

Recently, it has been shown that metformin increases the expression of the gene encoding the nuclear factor erythroid 2-related factor 2 (Nrf2) in the mouse heart [159]. It also has been proposed that Nrf2 protects against dyslipidemia-associated cardiovascular complications [160]. Nrf2 is a Cap‘n’Collar-basic leucine zipper protein (CNC-bZIP) family transcription factor [161] that plays an important role in protecting cells against environmental stress and maintaining redox homeostasis [162]. Nrf2 is involved in the regulation of several cellular processes, such as antioxidant defense, xenobiotic metabolism, protection against toxic metals, inhibition of inflammation, and repair of damaged proteins [163]. In addition to its main function (as a regulator of redox metabolism), Nrf2 is involved in the regulation of metabolism and mitochondrial bioenergetics [164,165,166]. As far as lipid metabolism is concerned, Nrf2 downregulates lipogenic enzymes, including ATP-citrate lyase, FASN and SCD1, in the liver of mice after genetic or pharmacological activation of the transcription factor [167]. On the other hand, the ATP-citrate lyase protein level in the liver is higher in Nrf2 knockout mice compared to wild-type mice [168]. These results suggest that Nrf2 can inhibit liver lipogenesis, via decreasing key lipogenic enzymes’ gene expression. This is consistent with the finding of greater lipid accumulation in the liver of Nrf2 knockout mice compared to wild-type ones [169,170,171]. Beyond downregulation of lipogenesis, Nrf2 enhances the efficiency of fatty acid oxidation [172,173,174], mainly via stimulation of long-chain fatty acid transport into mitochondria. It has been shown that human 293T cells with silenced Nrf2 have lower expression of CPT1, which plays a key regulatory role in long-chain fatty acid transport into mitochondria and fatty acid oxidation [173]. Furthermore, the CPT1 mRNA level is lower in the liver of Nrf2 knockout mice, compared to control animals (wild-type mice) [175]. The data presented above suggest that an increase in Nrf2 expression by metformin can lead to inhibition of fatty acid synthesis and stimulation of fatty acid oxidation. Inhibition of gene expression encoding lipogenic enzymes and stimulation of gene expression encoding CPT1 can lead to decreases in blood TAG concentrations (Figure 4). Based on the results obtained on animal experimental models, one can suppose that in diabetic patients treated with metformin an increase in Nrf2 level is taking place, which in turn exerts a beneficial effect on the blood lipid profile. However, this problem needs further study.

### 4.4. Metformin Reduces Blood Total Cholesterol and LDL-C Concentration via Suppressing ChREBP, Resulting in Decreased Gene Expression Encoding PCSK9

**Figure 5:**
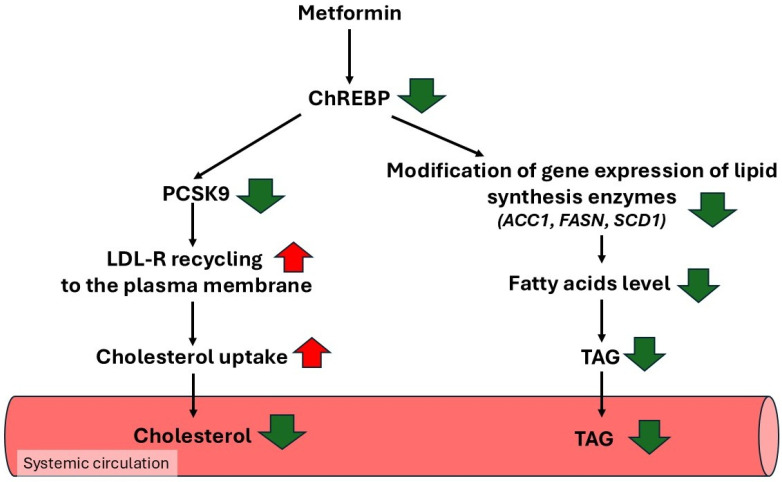
Metformin, via suppressing ChREBP, decreases plasma cholesterol and TAG concentrations. Diminished plasma concentration of cholesterol is a result of increased cholesterol uptake by the liver, due to inhibition by metformin of *PCSK9* expression; this leads to an increase in the LDL-R level. Lower plasma TAG concentration is the results of inhibition by metformin (via ChREBP) of lipogenic enzymes’ gene expression.

Metformin plays some role in protecting against the development of atherosclerotic cardiovascular disease (ASCVD) [176,177]. ASCVD is a major cause of mortality in patients with T2D [176,177]. Increased concentrations of blood LDL-C, a well-documented risk factor for ASCVD in diabetic and nondiabetic patients, were observed [99,178,179]. Statins, inhibitors of HMG-CoA reductase, a first line of lipid-lowering drugs, are widely used to lower blood LDL-C concentration and prevent atherosclerosis progression in both nondiabetic and diabetic patients [180]. Several papers also indicate that metformin treatment of diabetic patients effectively decreased blood LDL-C [99,121,178]. It is well documented that low-density lipoprotein receptor (LDL-R) plays a key regulatory role in blood LDL-C catabolism and consequently in plasma LDL-C concentration [181]. The physiological role of LDL-R is to transport LDL into cells. Upon the binding of LDL to LDL-R, the LDL-LDL-R complex internalizes via clathrin-mediated endocytosis [167]. Next, LDL is released by endosome acidification, a process that allows LDL-R recycling back to the cell membrane, while LDL is degraded in the lysosomes [181]. Increasing the level of LDL-R by statins, due to inhibition of HMG-CoA reductase and consequent reduction of cholesterol biosynthesis, leads to a significant decrease in blood cholesterol concentration [179]. Decrease in LDL-R on hepatocyte membrane, for instance in familial hypercholesterolemia [182], or in the case of elevated blood levels of PCSK9 [183,184], leads to hypercholesterolemia, a causative risk factor for cardiovascular disease. PCSK9—liver-derived protein [185] is an important drug target in hypercholesterolemia due to its ability to bind and mediate degradation of liver LDL-R, the most important receptor for clearance of blood LDL-C [186,187]. The ApoB100—the main protein component of LDL—binds to the ligand binding domain of LDL-R and undergoes acid-dependent release in early endosomes, allowing LDL-R to recycle to the cell surface [188,189]. In contrast, PCSK9 binds to the LDL-R domain with higher affinity than LDL [190]. Consequently, PCSK9 fails to release in early endosomes and directs LDL-R degradation in late endosomes/lysosomes [187,191,192]. These events lead to a significant decrease in LDL-R in the hepatocyte membrane and consequently to elevated circulating LDL-C concentration [193,194]. Administration of PCSK9 monoclonal antibody is associated with lower circulating LDL-C concentration [179,195,196]. Recently, Hu et al. [124] reported that metformin inhibits the expression of the gene encoding PCSK9 via blocking carbohydrate–responsive element binding protein (ChREBP) in an intracellular glucose and/or glucose metabolites (mainly glucose 6-phosphate)-dependent manner. Thus, metformin could significantly improve LDL-C homeostasis by inhibiting expression of the gene encoding PCSK9. A low blood level of PCSK9 enables the recycling of the LDL-R to the cell membrane. This leads to a higher number of LDL-R on the surface of hepatocyte membrane, an increase in cholesterol uptake, and consequently to a lower circulating cholesterol concentration (Figure 5).

The effect of metformin on blood LDL-C concentration resembles the effect of PCSK9 inhibitors. PCSK9 inhibitors—anti-PCSK9 monoclonal antibodies, e.g., Alirocumab, Evolocumab and Bococizumab—bind circulating PCSK9, and consequently prevent PCSK9-induced degradation of LDL-R [197]. The administration of PCSK9 inhibitors at clinically relevant doses to patients with cardiovascular diseases decreases blood LDL-C concentrations by approximately 50%, which is associated with significant risk reduction with respect to cardiovascular diseases [197,198]. Moreover, it has been shown that PCSK9 inhibitors improve blood TAG concentration (decrease by 10–20%), HDL-C concentration (increase by 5–10%) and Lp (a) concentration (decrease by 20–30%) [197]. The results presented above indicate that PCSK9 inhibitors significantly improve the blood lipid profile in patients with cardiovascular diseases. These beneficial effects on lipid profile may have a role in the reduction in cardiovascular disease. However, the potential mechanisms of PCSK9 inhibitors’ actions on serum TAG, HDL-C and Lp (a) concentrations are unknown. These problems are widely discussed in a review published in [197]. Recent studies indicate that metformin decreases blood Lp (a) levels in newly diagnosed diabetic patients; the mechanism of the drug’s action is unknown [199].

### 4.5. Metformin Reduces Blood TAG Concentration via Suppressing ChREBP, Resulting in Decreased Gene Expression Encoding Lipogenic Enzymes

ChREBP plays an important role in the regulation of lipid metabolism in the liver and adipose tissue [200,201,202,203]. It regulates fatty acid synthesis, fatty acid elongation and fatty acid desaturation via stimulation of transcription of genes encoding ACC1, FASN, elongase 6 and SCD1, respectively [203]. Moreover, it has been shown that single-nucleotide polymorphism of the ChREBP is associated with lipid disturbance and coronary heart disease [187,188,189,190,191,199,200,202,203,204]. Furthermore, in addition to cholesterol, plasma free fatty acid and triacylglycerol concentration in ChREBP^−/−^ mice fed a high-carbohydrate diet were significantly lower compared to the wild-type animals [205]. Thus, one can assume that metformin decreases ChREBP level, leading to decreases not only in plasma cholesterol, but also in free fatty acid and TAG concentrations. Figure 5 presents a scheme illustrating the proposed mechanistic hypothesis of metformin action on circulating cholesterol and triacylglycerol concentration in diabetic patients treated with the drug.

### 4.6. Metformin Reduces Blood Triacylglycerol and Cholesterol Concentrations via Suppressing Acetyl-CoA Synthetase, Which Plays a Key Role in Liver TAG and Cholesterol Biosynthesis from Acetate Formed by Gut Microbiota

**Figure 6:**
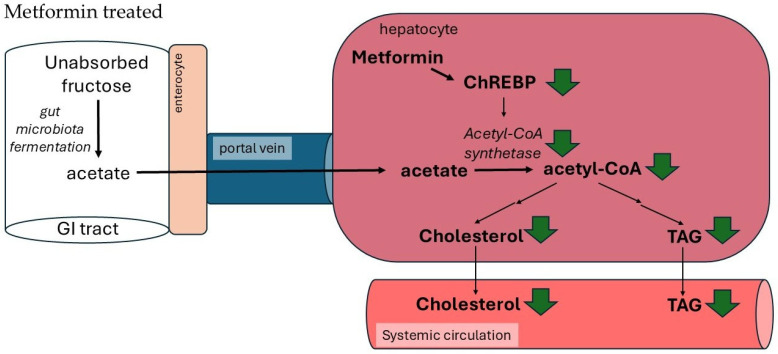
The effect of metformin on acetate (absorbed from intestines) metabolism in liver. In metformin-treated patients, acetate is slowly converted to acetyl-CoA due to lower activity of acetyl-CoA synthetase, which is inhibited via ChREBP by metformin. This leads to less cholesterol and TAG synthesis.

In humans, fructose, formed mainly from dietary sucrose or present in beverages and processed foods (as a corn syrup) [206], is absorbed in the intestine, transported via the portal vein to the liver, and metabolized. High fructose intake contributed to increasing rates of obesity and non-alcoholic fatty liver disease [207]. Unabsorbed fructose in the colon is fermented by the gut microbiota to acetate and other short-chain fatty acids [208,209,210,211,212,213]. Acetate formed by gut microbiota is transported via the portal vein to the liver, where it is converted by acetyl-CoA synthetase to acetyl-CoA (according to the reaction acetate + HS-CoA + ATP → acetyl-CoA + AMP + PPi), a precursor of cholesterol and fatty acids–substrates for triacylglycerols synthesis (Figure 6). Depletion of the intestinal microbiota by antibiotic treatment significantly suppresses the liver lipogenesis from acetate formed from fructose, without impairing intestinal and liver fructose metabolism, or the induction of key liver lipogenic enzymes [208]. Moreover, the decrease in the acetyl-CoA synthetase mRNA level by siRNA significantly diminishes the acetyl-CoA production in the liver [208]. These data strongly suggest that the intestinal microbiome providing acetate, a precursor of acetyl-CoA, plays an important role in hepatic lipogenesis and cholesterol synthesis. Recently it has been shown that the ChREBP is involved in the regulation of the conversion of gut microbiota-produced acetate to acetyl-CoA by activating the gene encoding hepatic acetyl-CoA synthetase [208]. Thus, one can assume that in diabetic patients treated with metformin, which suppresses ChREBP gene expression, acetyl-CoA synthetase activity is much lower. Consequently, less acetate is converted to acetyl-CoA in the liver. As result, less cholesterol and fatty acids (substrate for triacylglycerol) are produced (Figure 6). This may explain, at least in part, the lowering effect of metformin on blood cholesterol and triacylglycerol concentration.

### 4.7. Inhibition of Angiopoetin-like 3 Protein (ANGPTL3) Gene Expression by Metformin—A Potential Mechanism for Lowering Plasma TAG Concentrations

Angiopoetin-like 3 protein (ANGPTL3) is the secretory protein structurally similar to angiopoietins, the important factors regulating angiogenesis [214]. ANGPTL3 induces angiogenesis via its binding to integrin αγβ3 [215]. Besides, ANGPTL3 also plays an important role in the regulation of plasma TAG concentrations [216,217,218,219]. This protein inhibits the activity of lipoprotein lipase (LPL), the enzyme attached to the endothelial cells, which catalyzes hydrolysis of triacylglycerol, present mainly in circulating very low-density lipoproteins (VLDL) and chylomicrons, to free fatty acid (FFA) and glycerol [220,221]. Formed glycerol is taken up by the liver, where it can be converted to glucose and/or lipids (mainly to TAG and phospholipids), whereas FFA are taken up mainly by skeletal muscle, heart, kidney and liver, and oxidized as energy substrate [222]. It has been shown that the N-terminal coiled coil domain present in the ANGPTL3 molecule is an inhibitor of LPL activity [223,224]. Genetic studies indicate that ANGPTL3 significantly affects lipoprotein metabolism [225,226,227]. Rare loss–of–function (LoF) mutations in the gene encoding ANGPTL3 are associated with significantly lower blood TAG concentrations [228,229,230,231], which is due to the lower ANGPTL3 blood concentration and consequently higher LPL activity, which catalyze TAG degradation in VLDL and chylomicrons [223,224].

Several papers indicate that ANGPTL3 significantly decreases LPL activity [216,218,227,232]. Mice overexpressing ANGPTL3 develop hipertriacylglycerolemia [233]. On the other hand, mice lacking ANGPTL3 have increased LPL activity and reduced triacylglycerol concentrations [216,218]. Moreover, humans with the two nonsense AGNPTL3 alleles had low plasma concentrations of cholesterol and TAG [229,234]. The results briefly discussed above suggest that enhanced ANGPTL3 blood levels, both in animal models and humans, correlate positively with increased serum TAG, and LDL-C. In this context, ANGPTL3 resembles the action of apolipoprotein CIII (apo CIII). Similar to ANGPTL3, Apo CIII is a well-known inhibitor of LPL [220,235]. Although it is not clear how Apo CIII inhibits LPL, there is no doubt that when it is elevated, increased blood TAG concentration in some pathologies has been observed [235]. For instance, chronic kidney disease (CKD) patients exhibit elevated levels of blood Apo CIII, which inhibits LPL activity and consequently leads to dyslipidemia, a risk factor for cardiovascular disease (CVD) [236,237]. Moreover, it has been shown that loss-of-function mutations of the Apo CIII encoding gene are associated with lower plasma Apo CIII concentration and lower risk of ASCVD [238,239,240,241,242]. Several studies indicated that elevated plasma concentration of Apo CIII correlates with higher plasma TAG concentration and higher risk of ASCVD and the progression of coronary artery disease (CAD) [240,241,242]. The results presented above suggest that Apo CIII is an important pharmacological target for managing dyslipidemia [241,242].

**Figure 7:**
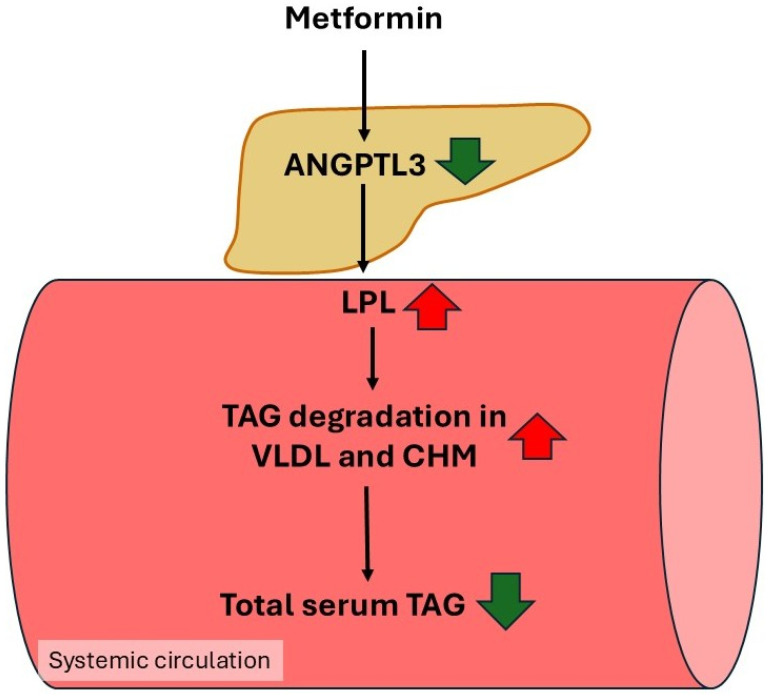
Metformin decreases angiopoetin-like 3 protein (ANGPTL3) expression, which increases the lipoprotein lipase (LPL) activity that catalyzes degradation of the TAG present in serum VLDL and chylomicrons.

It is interesting that in the last few years ANGPTL3 also garnered significant interest as a potential target for drugs lowering blood lipid concentration [242,243]. Special attention has been paid to monoclonal antibody evinacumab (Evkeeza) and antisense oligonucleotide strategies that significantly reduced plasma ANGPTL3 concentrations both in humans and in animal models, leading to reduction of plasma lipids [231,244,245]. In this context, the effect of metformin on the expression of the gene encoding ANGPTL3 looks interesting. Lin et al. [246], using HepG2 cells, a human hepatocyte cell line, found that metformin at a concentration of 0.5 mM inhibits approx. 40% expression of the gene encoding ANGPTL 3 in an AMPK-independent manner. These results suggest that inhibition of ANGPTL3 gene expression is a potential circulating lipid-lowering mechanism of metformin action. However, it cannot be guaranteed that the inhibition of the ANGPTL3 gene expression by a suprapharmacological concentration of metformin observed in tissue culture takes place in diabetic patients treated with clinically relevant dose of the drug. Nevertheless, the data presented above provides partial support for the concept that metformin may decrease plasma lipids, including TAG concentrations, and consequently provide cardiovascular benefit via inhibiting liver gene encoding ANGPTL3 (Figure 7).

Moreover, it has been shown that ANGPTL3 regulates plasma HDL-C concentrations via suppression of endothelial lipase (EL), which is synthesized mainly in endothelial cells and then fixed on the luminal surface of the endothelial cells by heparan sulphate proteoglycans [247]. It also has been shown that the plasma EL activity is significantly higher in coronary atherosclerosis and that it is inversely correlated with plasma high density lipoprotein (HDL) concentrations [248]. Some studies indicate that EL enhances inflammation [249] and increases the binding of monocytes to the endothelium [250]. It was also postulated that EL might be an independent risk factor for CAD [251]. Thus, it is tempting to speculate that metformin, via suppression of ANGPTL3, leads to lower blood triacylglycerol concentrations and lower EL activity. However, this effect of metformin on TAG concentrations and EL activity required further clarification.

### 4.8. Metformin as an AMPK Activator Enhances Reverse Cholesterol Transport

Wan et al. [252] found that metformin at a clinically relevant concentration activates the ATF1 (activating transcription factor 1) pathway in human monocyte-derived macrophages through AMPK. Based on these findings, Wan and coworkers suggest that metformin used at a pharmacological dose exhibits vascular protective effects. Atheroprotection is mediated by AMPK and ATF-1. ATF-1 shares extensive homology with cAMP response element binding protein (CREBP); like CREBP, it is expressed in many cell types and is capable of dimerizing with CREBP [253]. ATF-1 homodimers and ATF-1/CREBP heterodimers bind to the CRE response element and mediate transcriptional effects on protein kinase A (PKA) [253]. ATF1 directs atheroprotective macrophages via coordinated iron handling and lipid-laden macrophage (often called foam cells) protection [254,255]. It has been also shown that apoE^−/−^ mice treated with AMPK activators, including metformin, show enhanced reverse cholesterol transport [256], a process by which the human body removes excess cholesterol from peripheral organs and delivers cholesterol to the liver, where it is redistributed (mainly via formed VLDL) to other organs and/or converted to bile acid, which is removed to the intestine via the bile duct. These authors found that AMPK activation by metformin led to elevated expression of genes encoding ATP-binding cassette A1 (ABCA1) and ATP-binding cassette G1 (ABCG1) in macrophages and scavenger receptor class B type 1 (SR-B1) and lecithin cholesterol acyltransferase (LCAT) in the liver [256]. ABCA1 and ABCG1 play an important role in cholesterol efflux from macrophages to HDL, thereby reducing cholesterol levels in macrophages [257,258]. Theoretically, the increase in ABCA1/ABCG1 expression by metformin in macrophages increases the ability of these cells to efflux cholesterol, which is then incorporated into HDL or binds to ApoA1 and then is transported to the liver. The SR-B1 is responsible for the selective uptake of HDL-derived cholesteryl esters into the cell. HDL-Cl binds to SR-B1 on the cell surface, and cholesteryl esters are delivered to the cell without internalization of the entirety of the HDL particles [259]. LCAT is an HDL-associated enzyme (pro teryl ester + lysolecithin (lisophosphatidylcholine) [260]. Via increases in HDL-cholesterol esterification, LCAT is an important enzyme in HDL metabolism and likely enhances reverse cholesterol transport [260]. ABCA1/ABCG1, SR-B1 and LCAT are important elements of reverse cholesterol transport. These results suggest that metformin, like other AMPK activators, reduces atherosclerosis. It also has been shown that macrophages obtained from Ampkβ1^−/−^ mice have more lipid accumulation and lower cholesterol efflux, whereas Ampkβ1 activation by salicylate significantly improves macrophage cholesterol homeostasis (i.e., decrease in cholesterol accumulation and an increase in cholesterol efflux) and foam cell formation [261]. These data support the regulatory role of AMPK in mediating the reverse transport of cholesterol. Since AMPK β1 is the predominant subunit in human macrophages [262], it is tempting to speculate that the activation by metformin of macrophage AMPK might mediate, at least in part, the beneficial effect of this drug on blood lipid concentration and consequently on cardiovascular disease in diabetic patients. The important last step in the reverse transport of cholesterol is mediated by the cholesterol transporters ABCG5 (ATP binding cassette transporter G5) and ABCG8 (ATP binding cassette transporter G8) [263,264,265]. These transporters are located on the canalicular membrane of hepatocytes and are responsible for the excretion of cholesterol into the liver bile duct. It has been shown that single nucleotide polymorphisms (SNPs) in the genes encoding ABCG5/ABCG8 are associated with elevated blood total cholesterol, LDL-cholesterol concentrations, and with coronary artery disease [266,267,268]. Molusky et al. [269] found that metformin at a concentration 0.5 mM increases ABCG5 and ABCG8 mRNA levels in mouse primary hepatocytes. Furthermore, they found that metformin increases expression of the bile salt transporter (bsep). The bsep (in animals) and BSEP (in humans) pumps (encoded by abcb11 and ABCB11 respectively), play an important role in the canalicular excretion of monovalent bile salt into the intestine via the bile duct [270]. Given the fact that bile acids are formed from cholesterol, the BSEP pump is an important element of the route of elimination of part of cholesterol from the human body.

**Figure 8:**
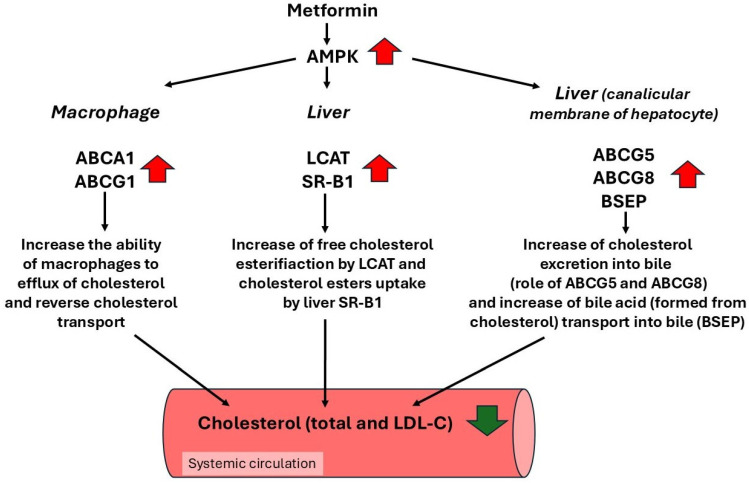
AMPK activated by metformin decreases serum total and LDL-cholesterol concentrations via stimulation of reverse cholesterol transport.

The results briefly presented above indicate that metformin via activation of AMPK increases expression of several important elements of the reverse cholesterol transport system, including: ABCA1 and ABCG1 in macrophages; and SR-B1, LCAT, ABCG5, ABCG8, and BSEP in the liver. As a consequence of these events, cholesterol is more efficiently removed from peripheral tissue including macrophages and transported by HDL-C particles to the liver for excretion via bile ducts into the intestine as free cholesterol and/or bile acid formed from cholesterol (Figure 8). Consequently, metformin might exert a positive effect on blood lipid concentration, especially on total and LDL-C concentrations in diabetic patients. However, one must remember that while some effects described above are observed at a clinically relevant concentration of metformin (approx. 10 µM), other processes require suprapharmacological concentrations of the drug (0.5 mM).

### 4.9. Metformin May Have a Beneficial Effect on Dyslipidemia in T2D Patients via Activation of AMPK, Which Inhibits LXRα Activity

Liver X receptor alfa (LXRα), encoded by NR1H2, is a nuclear receptor playing an important role in the regulation of lipid metabolism [271]. Its activation increases liver and blood TAG and LDL-C concentrations both in animal experimental models [272,273] and in humans [274]. The LXRα-dependent upregulation of lipogenic enzymes and inhibition of LPL activity by increasing ANGPTL3 and Apo CIII were found to be mainly responsible for the increases in liver and blood TAG and LDL-C levels [275]. Inhibition of liver LXRα activity significantly reduces lipogenesis and, consequently, blood TAG concentration [276]. Importantly, therapies that inhibit LXRα target genes involved in lipogenesis and lipid clearance show promising effects in clinical trials [277]. Furthermore, it has been shown that activation of AMPK inhibits ligand-induced LXR activity on lipogenic enzymes [278,279]. It also has been shown that metformin reduces liver TAG level, and this process involves the AMPK/LXRα signaling pathway [280]. Moreover, it also has been reported that metformin inhibits liver lipogenesis via selective inactivation of LXRα [281]. The results suggest that metformin, via activation of AMPK, which inhibits LXRα activity, may have a beneficial effect on hyperlipidemia in T2D patients.

### 4.10. Serum Lipid Alterations After 1 Dose of Metformin Intake by Healthy Subjects Analyzed by Mass Spectrometry

Recently serum lipid alteration after one dose (500 mg) of metformin intake by 26 healthy subjects (age 18–50 years) was investigated using mass spectrometry [282]. The authors of this paper found that metformin increased 33 and decreased 192 lipid compound concentrations in the sera of examined subjects. Among the significantly altered lipids concentrations by drug were compounds involved in (a) arachidonic acid metabolism, (b) steroid hormone biosynthesis and (c) glycerophospholipids biosynthesis. Furthermore, among the lipids significantly altered by metformin were chemical compounds involved in lipid signaling pathways. For instance, sphingosine 1-phosphate is a regulator of many physiological processes and, when dysregulated, contributes to some diseases including atherosclerosis, diabetes, cancers and autoimmune disorders [283]. According to the authors, the lipids, altered by metformin, could be associated with diet and physical activity and the pleiotropic effects of the drug in patients with (a) T2D, (b) insulin resistance, (c) lipid disturbance and cardiovascular disease and (d) some cancers [282]. The same scientific group examined a metabolic pattern in healthy subjects given a single dose of metformin (500 mg). They found that 36 metabolites involved in several biochemical processes were dysregulated after metformin treatment [284]. The most-altered by metformin were metabolites of branched-chain amino acids (BCAAs) [284]. However, other studies indicate that the effect of metformin on the concentrations of blood BCAAs in diabetic and non-diabetic patients is inconclusive (for details see review [285]).

## 5. Conclusions

Diabetic dyslipidemia is usually characterized by elevated blood triacylglycerol concentrations, low HDL-C concentrations and high concentrations of small-dense LDL particles. This lipid pattern is particularly atherogenic and strongly associated with microvascular and macrovascular complications.

Metformin treatment of T2D patients not only leads to decreases in blood glucose concentrations but also to decreases in blood T-C, LDL-C and TAG concentrations. Some authors show that blood HDL-C concentrations are elevated by metformin. Most of these beneficial effects of metformin were observed at a clinically relevant dose of the drug.

Several molecular mechanisms are potentially involved in the beneficial effects of metformin on blood lipid profile, including: (a) activation of AMPK by clinically relevant concentrations of metformin; (b) promotion by metformin of VLDL-triacylglycerol clearance by brown adipose tissue; (c) increase in Nrf2 expression by metformin; (d) inhibition of expression of genes encoding PCSK9 by metformin; (e) down-regulation of hepatocyte ChREBP by metformin, which leads to reductions in liver triacylglycerol and cholesterol synthesis from acetate formed by gut microbiota; (f) stimulation of reverse cholesterol transport by metformin; (g) activation of AMPK, which inhibits LXRα activity and ultimately affects dyslipidemia; and (h) inhibition of ANGPTL3 gene expression by metformin observed at suprapharmacological doses of the drug.

Furthermore, the data suggest that metformin can affect lipid metabolism independently of changes in blood glucose concentrations.

Overall, the data presented in this review indicate that metformin significantly improves dyslipidemia, reducing cardiovascular risk in patients with T2D. However, further studies are needed to evaluate and validate the beneficial role of metformin treatment of T2D patients with reference to lipid compounds. We also need to remember metformin’s side effects, including diarrhea, nausea, and vomiting. Notably, though, these GI side effects often diminish over time and can be minimized by careful dose adjustments and taking metformin at mealtimes [286].

## Data Availability

No new data were created.

## Abbreviations

The following abbreviations are used in this manuscript:

ABCA1	ATP-binding cassette A1
ABCG1	ATP-binding cassette G1
ABCG5	ATP-binding cassette transporter G5
ABCG8	ATP-binding cassette transporter G8
ACC1	Acetyl-CoA carboxylase isoform I
ACC2	Acetyl-CoA carboxylase isoform II
ADP	Adenosine diphosphate
AMP	Adenosine monophosphate
AMPK	AMP-activated protein kinase
ANGPTL3	Angiopoietin-like 3 protein
APH-1	Anterior pharynx defective 1
Apo CIII	Apolipoprotein CIII
ApoB100	Apolipoprotein B100
APOE3	Apolipoprotein E3
ASCVD	Atherosclerotic cardiovascular disease
ATF1	Activating transcription factor 1
ATP	Adenosine triphosphate
ATP6AP1	ATPase H+ Transporting Accessory Protein 1
AXIN	Axis inhibitor
BAT	Brown adipose tissue
BCAA	Branched-chain amino acids
BSEP	Bile salt transporter
bZIP	Basic leucine zipper protein
CAD	Coronary artery disease
CaMKK2	Ca^2+^/calmodulin dependent protein kinase 2
cAMP	Cyclic adenosine monophosphate
CETP	Cholesteryl ester transfer protein
CHL	Cholesterol
ChREBP	Carbohydrate response element binding protein
CKD	Chronic kidney disease
CNC	Cap‘n’Collar transcription factor
COVID-19	Coronavirus disease 2019
CPT1	Carnitine palmitoyl-CoA transferase 1
CRE	cAMP Response Element
CREBP	cAMP response element binding protein
CVD	Cardiovascular disease
CYPs	Cytochrome P450 enzymes
EL	Endothelial lipase
FASN	Fatty acids synthase
FFA	Free fatty acids
GDF15	Growth differentiation factor 15
GIP	Glucose-dependent insulinotropic peptide
GLP-1	Glucagon-like peptide 1
GSH	Reduced glutathione
GSSG	Oxidized glutathione
HDL	High density lipoprotein
HDL-C	High density lipoprotein cholesterol
HMG-CoA	3-hydroxy-3-methylglutaryl-CoA
HSL	Hormone-sensitive lipase
Km	Michaelis–Menten constant
LCAT	Lecithin cholesterol acyltransferase
LDL	Low density lipoprotein
LDL-R	Low density lipoprotein receptor
LKB1	Liver kinase B1
LoF	Loss-of-function mutations
Lp(a)	Lipoprotein a
LPL	Lipoprotein lipase
LXRα	Liver X receptor alfa
M	Metformin
MATE-1	Multi-drug and toxin extrusion-1 transporter
MATE2-K	Multi-drug and toxin extrusion2-K transporter
mGPDH	Mitochondrial glycerol 3-phosphate dehydrogenase
NAD	Nicotinamide adenine dinucleotide
NADH	Nicotinamide adenine dinucleotide reduced
NAFLD	Non-alcoholic fatty liver disease
NASH	Non-alcoholic steatohepatitis
NKX3.1	Androgen regulated gene encoding transcription factor
Nrf2	Nuclear factor erythroid 2-related factor 2
OCT1	Organic cation transporter 1
OCT2	Organic cation transporter 2
OCT3	Organic cation transporter 3
OCTN1	Carnitine/organic cation transporter
PCSK9	Proprotein convertase subtilisin/kexin 9
PEN2	Presenilin enhancer 2
Pi	Phosphate
PKA	Protein kinase A
PMAT	Plasma membrane monoamine transporter
PPi	Pyrophosphate
Rap 1	Ras-related protein 1
SCD1	Stearoyl-CoA desaturase 1
SERT	Serotonin reuptake transporter
SGLT2	Sodium/glucose cotransporter 2
SLC19A3	Solute carrier family 19 member 3
SLC22A1	Solute carrier family 22 member 1
SLC22A4	Solute carrier family 22 member 4
SLC29A4	Solute carrier family 29 member 4
SLC6A4	Solute carrier family 6 member 4
SR-B1	Scavenger receptor class B type 1
SREBP-1c	Sterol regulatory element-binding protein 1c
SREBP2	Sterol regulatory element-inding protein 2
STK 11 (AMPK)	Serine/threonine kinase 11
T2DM	Type 2 diabetes
TAG	Triacylglycerol
THTR-2	Human thiamine transporter 2
v-ATPase	Vacuolar adenosine triphosphatase
VLDL	Very low-density lipoprotein
